# Impact of Climate Change on the Relict Tropical Fish Fauna of Central Sahara: Threat for the Survival of Adrar Mountains Fishes, Mauritania

**DOI:** 10.1371/journal.pone.0004400

**Published:** 2009-02-10

**Authors:** Sébastien Trape

**Affiliations:** 1 Institut de Recherche pour le Développement (IRD), Unité de Recherche 070, Dakar, Sénégal; 2 Laboratoire ECOLAG, Unité Mixte de Recherche 5119, Université Montpellier II, Montpellier, France; University of Utah, United States of America

## Abstract

**Background:**

Four central Sahara mountainous massifs provide habitats for relict populations of fish. In the Adrar of Mauritania all available data on the presence and distribution of fish come from pre-1960 surveys where five fish species were reported: *Barbus pobeguini*, *Barbus macrops*, *Barbus mirei*, *Sarotherodon galilaeus,* and *Clarias anguillaris*. Since 1970, drought has had a severe impact in the Adrar where rainfall decreased by 35%. To investigate whether the relict populations of fish have survived the continuing drought, a study was carried out from 2004 to 2008.

**Methodology/Principal Findings:**

An inventory of perennial bodies of water was drawn up using a literature review and analysis of topographical and hydrological maps. Field surveys were carried out in order to locate the bodies of water described in the literature, identify the presence of fish, determine which species were present and estimate their abundance. The thirteen sites where the presence of fish was observed in the 1950s -Ksar Torchane, Ilij, Molomhar, Agueni, Tachot, Hamdoun, Terjit, Toungad, El Berbera, Timagazine, Dâyet el Mbârek, Dâyet et-Tefla, Nkedeï- were located and surveyed. The Ksar Torchane spring -type locality and the only known locality of *B. mirei*- has dried up at the height of the drought in 1984, and any fish populations have since become extinct there. The Timagazine, Dâyet el Mbârek and Dâyet et-Tefla pools have become ephemeral. The Hamdoun guelta appears to be highly endangered. The fish populations at the other sites remain unchanged. Four perennial pools which are home to populations of *B. pobeguini* are newly recorded.

**Conclusion/Significance:**

The tropical relict fish populations of the Adrar mountains of Mauritania appear to be highly endangered. Of thirteen previously recorded populations, four have become extinct since the beginning of the drought period. New fish population extinctions may occur should low levels of annual rainfall be repeated.

## Introduction

The presence of fish in the Sahara, evidence of the world's hottest desert humid past during the Holocene period, was recognised in the first Saharan exploration expeditions in the early 20th century [1]–[4]. The work undertaken in the 1950s expanded the inventory of perennial bodies of water -springs, gueltas or pool - which are home to this relict fauna [5]–[8]. More recently, systematic revisions, especially for the *Barbus* genus, and re-examination of the collected materials, have clarified the species identification and biogeographical affinities of the fish species present in the Sahara [9], [10].

Four of the six main central Saharan mountainous massifs are home to fish: the Ahaggar and Tassili-n-Ajjer in southern Algeria, the Adrar in Mauritania and the Tibesti in Chad [3]–[10]. There are no fish in the Aïr massif of Niger and the Iforas massif of Mali. On the southern fringes of the Sahara, relict fish fauna also survive in the Tagant of Mauritania and in the Ennedi of Chad. In total, sixteen species and sub-species of fish have managed to survive in the central Sahara, most commonly in a very low number of watering places, sometimes in a single spring or guelta (Table 1). These species are the following: *Barbus macrops* Boulenger, 1911, *Barbus pobeguini* Pellegrin, 1911, *Barbus deserti* Pellegrin, 1909, *Barbus bynni occidentalis* Boulenger, 1911, *Barbus callensis biscarensis* Boulenger, 1911, *Labeo parvus* Boulenger, 1902, *Labeo niloticus* (Forsskål, 1775), *Raiamas senegalensis* (Steindachner, 1870), *Clarias anguillaris* (Linnaeus, 1758), *Clarias gariepinus* (Burchell, 1822), *Epiplatys spilargyreius* (Duméril, 1861), *Hemichromis bimaculatus* Gill, 1862, *Sarotherodon galilaeus galilaeus* (Linnaeus, 1758), *Sarotherodon galilaeus borkuanus* (Pellegrin, 1919), *Astatotilapia desfontainesi* (Lacépède, 1803) and *Tilapia zillii* (Gervais, 1948) [3]–[5], [8], [10].

**Table 1:** Relict fish species of Central Sahara: distribution in Sahara and adjacent Sahelian basins.

Family and species	Distribution	
	Regions of Sahara	Adjacent basins
Cyprinidae		
*Barbus c. biscarensis*	Mouydir/Ahaggar/Tassili	Atlas wadis (Maghreb)
*Barbus b. occidentalis*	Tibesti	Senegal/Niger/Chad
*Barbus deserti*	Tassili	Endemic
*Barbus macrops*	Adrar/Ahaggar/Tibesti	Senegal/Niger/Chad
*Barbus pobeguini*	Adrar	Senegal/Niger
*Labeo niloticus*	Tibesti	Nile
*Labeo parvus*	Tibesti	Senegal/Niger/Chad
*Raiamas senegalensis*	Tibesti	Senegal/Niger/Chad
Clariidae		
*Clarias anguillaris*	Adrar	Senegal/Niger/Chad/Nile
*Clarias gariepinus*	Tassili/Tibesti	Senegal/Niger/Chad/Nile
Cyprinodontidae		
*Epiplatys spilargyreius*	Borkou	Senegal/Niger/Chad/Nile
Cichlidae		
*Hemichromis bimaculatus*	Tassili/Borkou	Senegal/Niger/Chad/Nile
*Sarotherodon g. galilaeus*	Adrar	Senegal/Niger/Chad/Nile
*Sarotherodon g. borkuanus*	Tibesti/Borkou	Endemic
*Astatotilapia desfontainesi*	Mouydir	Atlas wadis (Algeria/Tunisia)
*Tilapia zillii*	Mouydir/Ahaggar/Tassili/Tibesti	Senegal/Niger/Chad/Nile

The species richness and affinities of the relict populations of Saharan fish differ significantly depending on the massif. In the Ahaggar, three species are present, including one Paleoarctic species, *B. c. biscarensis*, and two Afrotropical species, *B. macrops* and *T. zillii*. In the Tassili-n-Ajjer and its Mouydir extension, the relict fauna comprises seven species: *B. deserti*, *B. c. biscarensis* and *A. desfontainesi*, which are Paleoarctic; and *C. gariepinus*, *C. anguillaris*, *H. bimaculatus* and *T. zillii*, which are Afrotropical. In the Adrar of Mauritania, four Afrotropical species are present: *B. pobeguini*, *B. macrops*, *S. g. galilaeus* and *C. anguillaris*. In the Tibesti and its Borkou extension, there are nine Afrotropical species: *B. macrops*, *B. bynni occidentalis*, *L. parvus*, *L. niloticus*, *R. senegalensis*, *C. gariepinus*, *E. spilargyreius*, *S. galilaeus borkuanus* and *T. zillii*.

**Figure 1:**
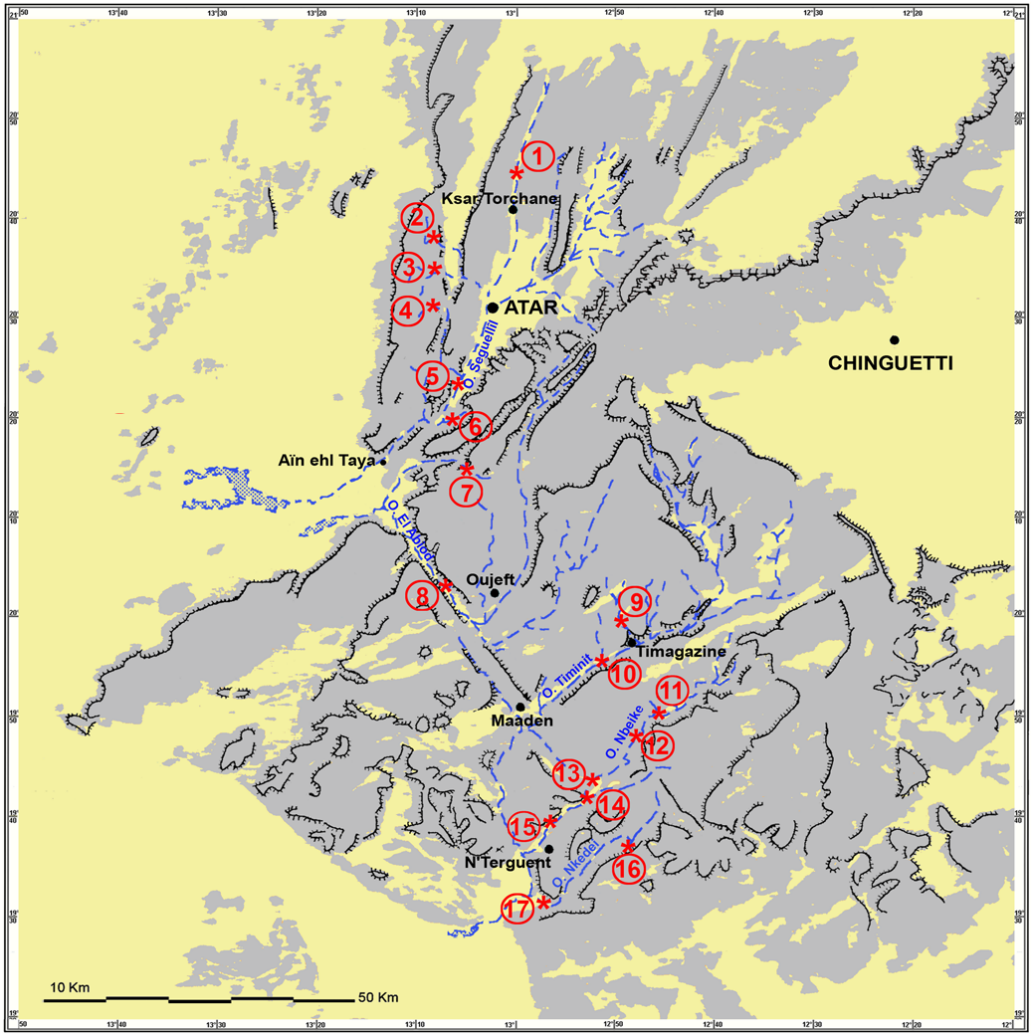
Map of Adrar mountains, Mauritania. Rocky areas are shown in grey, sandy areas in yellow. Numbers indicate gueltas, ponds and springs where the presence of fish was reported in the literature and/or observed during the present study. 1 : spring of Ted at Ksar Torchane ; 2 : Ilîj guelta ; 3 : Molomhar gueltas ; 4 : Agueni ponds ; 5 : Tachot guelta ; 6 : Hamdoun guelta ; 7 : Terjit springs ; 8 : Toungad guelta ; 9 : El Berbera guelta ; 10 : Timagazine wadi pond ; 11 : Dâyet el Mbârek pond ; 12 : Agmeïmine guelta ; 13 : Dâyet et-Tefla pond ; 14 : Douâyât Lemgasse pond ; 15 : Dâyet Legleïg pond ; 16 : Nkedeï guelta and pond ; 17 : Glât el Bil pond.

The presence of fish in the Adrar mountains of Mauritania has been known since 1913, when Pellegrin first mentioned the specimens of *C. senegalensis* ( = *C. anguillaris*) and *T. galilaea* ( = *S. galilaeus*) collected by Chudeau near Atar [1]. In 1937, Pellegrin added *B. pobeguini* and *B. deserti* ( = *B. macrops*) collected by Monod in various Adrar gueltas [3]. Between 1951 and 1955, Monod published three detailed papers on the freshwater fish species of Mauritania [5]–[7]. These papers are still the only sources of original data on the inventory and population of water bodies which provide habitats for fish in the Adrar region (Figure 1). They indicated the presence of *B. deserti* ( = *B. macrops*) in Ilîj, Agueni and Tachot (Seguellîl wadi basin) and in the Toungad guelta (El Abiod wadi basin), the presence of *C. senegalensis* ( = *C. anguillaris*) in the same gueltas, with the exception of those in Ilîj and Tachot, the presence of *T. galilaea* ( = *S. g. galilaeus*) in Molomhar and of *B. pobeguini* in all the above mentioned gueltas and in the Ksar Torchane spring (Tengharâda wadi, a tributary of the Seguellîl wadi), in the Terjit springs (Terjit wadi, a tributary of the El Abiod wadi) and in several gueltas or pools in the three southern Adrar basins which are completely separate from the above basins: in El Berbera and Timagazine (Timagazine-Timinit wadi basin), Dâyet el Mbârek and Dâyet et-Tefla (Nbéïké wadi) and Nkedeï (Nkedeï wadi). In 1952, Estève [11] described a new subgenus and species of *Barbus* in the Ksar Torchane spring: *B. (Hemigrammocapoeta) mirei*, characterised by an incomplete lateral line. However, their validity was doubted by Monod [6] and not recognised by Lévêque who considered *B. mirei* as synonym of *B. pobeguini* [10]. Additional data on the aquatic fauna of the Adrar region have been provided by Villiers [12] and Dékeyser & Villiers [13]. Reviews of Saharan aquatic fauna, including the Adrar region, have been carried out by Dumont [14], [15], Lévêque [10] and Le Berre [16].

Since 1970, a drought of historically unprecedented proportions persists in West Africa, with a reduction in annual recorded rainfall of 20 to 30% in the Sudanese savannah and up to 40% in the Sahel region on the southern fringes of the Sahara [17]–[20]. The rainfall deficit was also very marked in the Adrar region, with the average recorded rainfall in Atar decreasing by 35%, from 106.8 mm in 1921–1969 to only 69.8 mm in 1970–2007 [21]. Furthermore, some years were almost completely dry, with recorded rainfall in Atar of less than 20 mm in 1971, 1977, 1982 and 1996 and only 7 mm in 1983, which could be insufficient to sustain the few perennial bodies of water in the Adrar region. As all available data on the presence and distribution of fish comes from pre-1960 surveys, it became important to investigate to what extent these fish populations were able to resist such a long, intense period of drought. In order to assess the current state of ichthyologic fauna, a series of surveys was carried out from 2004 to 2008. The results of these surveys show that the relict fish fauna of Adrar mountains is highly endangered and that several extinctions of fish populations has already occurred.

## Materials and Methods

### Study area

The Adrar is a low central massif located between 19°–21° N and 11°–14° W, with as its highest point the 850 m peak of Teniagouri. It is mainly composed of a series of sandstone plateaux, edged by steep cliffs, which are located between 200 m and 600 m in altitude. To the west, the Adrar dominates the sandy or rocky plains of the Inchiri and Amsaga, which lie at around 100 metres above sea level. To the east and north, it is bordered by the Majâbat, Ouarane and Oumm Aghouäba dunes. To the south, it is separated from the Tagant plateaux by the Khat depression. The hydrographic network of the Adrar comprises a high number of fossil valleys, where the water only flows on a few days of the year, most often in August or September, when there is sufficient rainfall. However, despite the scarcity of rainfall, the sandstone nature of the relief allows the water to permeate and perennially seep into the various gueltas and pools, creating the conditions for the survival of a relatively rich fauna of relict fish despite the gradual drying of the Sahara since the Holocene period [22], [23].

### Methods

During a first phase an inventory of the perennial or semi-perennial bodies of water in the Adrar region of Mauritania -both with or without reported presence of fish- was drawn up from a literature review and an analysis of topographical, geological and hydrological maps of the region.

In a second phase, a series of field surveys were carried out between October 2004 and June 2008 in order to locate the bodies of water mentioned in the literature and to look for the presence of fish, identify the species present and, where possible, estimate their abundance. A search for fish in perennial bodies of water where their presence was not mentioned in the literature was also carried out. Whenever local populations were present near watering places, they were also interviewed about the presence of fish.

The fish were captured using landing nets, nets or lines. In most cases they were returned to the water after being identified, but vouchers of each species and locality were kept and their identification verified using the keys drawn up by Lévêque [24], Teugels [25] and Teugels & Thys Van den Audenaerde [26]. Sub-aquatic visual explorations were also carried out at Ksar Torchane, Ilîj, Molomhar, Agueni, Tachot, Hamdoun, Terjit and El Berbera.

The study was approved by the review board of the IRD special programme “Action Thématique Interdépartementale Evolution Climatique et Santé”.

## Results

All bodies of water where the presence of fish had been indicated were identified, located in the field and surveyed. Several pools and gueltas not previously mentioned in the literature were also surveyed. The report on these surveys follows:

### Ksar Torchane (spring)

**Figure 2:**
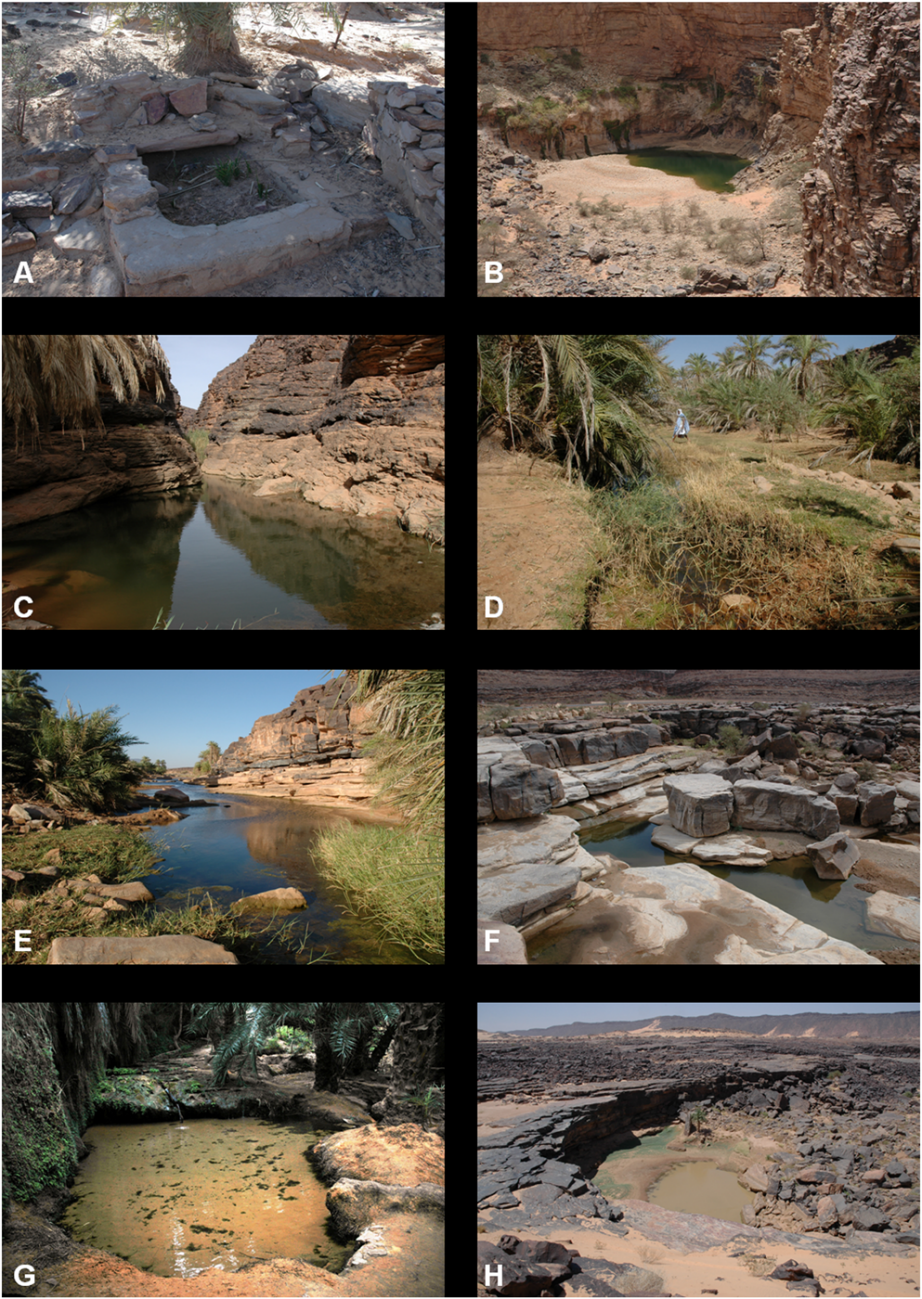
Gueltas and springs from the Seguellîl wadi basin. A: The former spring of Ted at Ksar Torchane; B: Ilîj guelta.; C: The first guelta of Molomhar; D: Pond at Agueni; E: Tachot guelta; F: Hamdoun guelta; G: Terjit springs; H: Toungad guelta.

The Ksar Torchane spring (20°43′654 N/13°00′593 W) is located in the hamlet of Ted, on the edge of the Tengharâda wadi, a tributary of the Seguellîl wadi, at the northern limit of the Ksar Torchane palm grove (Figure 2A). It is the most northerly of all the sites where freshwater fish have been reported in Mauritania. The fifteen specimens of the type series of *B. (Hemigrammocapoeta) mirei* Estève, 1952, came from this spring. The presence of *B. pobeguini* has also been reported [5].

The Ted spring was continually dry during the surveys carried out in October 2004, April 2005, October 2006, April 2007 and May 2007. The well in the bed of the wadi which was previously fed by the spring was flowing at the time of each survey but no fish were observed, neither from the surface nor during a sub-aquatic exploration in April 2007. The height of the water was then 2.5 m, with a water volume of 12 m^3^. According to the primary school teacher from Ted, who is originally from the hamlet, the fish disappeared in early 1984 when the spring and the well both ran dry for the first time and a vast cement basin adjoining the spring was also found dry. The fort overlooking the spring was at the time the headquarters of a small military garrison which enlarged the well using explosives in 1984 before having to definitively leave the site that same year. The well then continued to dry up, and new enlargement works using explosives were carried out in 1990.

### Ilîj (guelta)

The Ilîj guelta (20°38′046 N, 13°08′490 W) is located at the foot of a cliff in the canyon of the Ilîj wadi, a tributary of the Seguellîl wadi (Figure 2B). The presence of *B. pobeguini* and *B. macrops* was reported [5].

The guelta was flowing at the time of the surveys in April and May 2007 and water was seeping from the cliff at a number of locations. Several thousand *B. macrops* and *B. pobeguini* were present. The freshwater jellyfish *Limnocnida tanganyicae* was observed.

### Molohmar (gueltas)

The three perennial Molohmar gueltas are several dozen metres apart in a narrow canyon in the Oumm Lemhar wadi, a tributary of the Ilîj wadi, which they block completely (20°35′229 N, 13°08′794 W) (Figure 2C). They have the greatest species richness, with four species reported: *B. pobeguini*, *B. macrops*, *C. anguillaris* and *S. g. galilaeus* [5], [12], [13].

In the first, and deepest, guelta, the water was six metres deep in May 2007. Several thousand *B. pobeguini*, *B. macrops* and *S. g. galilaeus* were present in each. *C. anguillaris* and *L. tanganyicae* were also observed.

### Agueni (palm grove pools)

The Agueni palm grove is located in the bed of the Taghadem wadi, a tributary of the Seguellîl wadi. It comprises several small perennial pools fed by infiltration through the sandy soil (Figure 2D). These pools are known to be home to three species of fish: *B. pobeguini*, *B. macrops* and *C. anguillaris* [5].

Sixteen small pools were flowing in June 2008 at the foot of the palm groves, over a distance of around 300 m (upstream pool: 20°31′380 N, 13°08′714 W; downstream pool: 20°31′326 N, 13°08′556 W). The largest pool was 10 m long and 3 m wide; the deepest reached a depth of one metre. *B. pobeguini* and *B. macrops* were observed in most of the pools surveyed. *C. anguillaris* was not observed; however, according to the residents of the palm grove, it is still abundant. An ephemeral downstream guelta (20°31′204 N, 13°08′207 W) was around 100 m long in September 2007, but only 3 m long in April 2007. It was also home to *B. pobeguini* and *B. macrops*.

### Tachot (guelta)

The Tachot guelta (20°24′410 N, 13°06′465 W) is located at the foot of a cliff in the bed of the Seguellîl wadi. Monod had observed barbs there without being able to capture them and had presumed that they were *B. pobeguini* [5].

The water surface of this vast guelta was around 300 m^2^ and reached a maximum depth of 50 cm in April 2007 (Figure 2E). Around 3,000 Barbs were present, around a third of which were *B. macrops* and two-thirds were *B. pobeguini*.

### Hamdoun (guelta)

The Hamdoun guelta (20°19′380 N, 13°08′550 W) is located in the Fârech wadi, at the foot of a small cliff, several metres below the main Atar-Nouakchott road (Figure 2F). The presence of *B. pobeguini*, *B. macrops* and *C. anguillaris* was reported [5], [12], [13]. Other gueltas are located downstream, spread over a distance of around one kilometre, down to the confluence with the Seguellîl wadi, but all are ephemeral. Upslope of the road, there are also several gueltas where the presence of fish has been reported despite the gueltas being ephemeral [5], [12].

Eleven surveys were carried out between October 2004 and June 2008. *B. pobeguini*, *B. macrops* and *C. anguillaris* were regularly observed in the perennial guelta and in several of the ephemeral downstream gueltas when they were flowing. No fish were ever observed in the gueltas above the road which overlooks the perennial guelta. In October 2004, October 2006, September 2007 and November 2007, the Fârech wadi flowed over the road and into the perennial guelta after falling for four metres. It was in July 2007 that the lowest water level was measured in the perennial guelta: its maximum depth was 35 cm and the volume of water was only 1.7 m^3^. The water was too cloudy to measure the fish population accurately, but it was certainly lower than 200 specimens (240 barbs and seven catfish were counted two months previously when the volume of water was 4 m^3^).

### Terjit (springs)

The Terjit springs (20°15′045 N, 13°05′200 W), one hot (32°C) and the other cold, are several metres apart in a gorge and form a small stream which flows around a hundred metres before filtering into the sand (Figure 2G). Only the presence of *B. pobeguini* was reported [5], [12], [13].

*B. pogeguini* was present on each occasion in small numbers (probably under 300 specimens) at the four surveys that were carried out between October 2006 and May 2007.

### Toungad (guelta)

The Toungad guelta (20°03′771 N, 13°07′263) is located at the foot of a cliff in the El Abiod wadi basin (Figure 2H). The presence of *B. pobeguini*, *B. macrops* and *C. anguillaris* was reported [7].

Only *B. pobeguini* was observed in a rapid survey in April 2005 when the water was cloudy.

### El Berbera (guelta)

**Figure 3:**
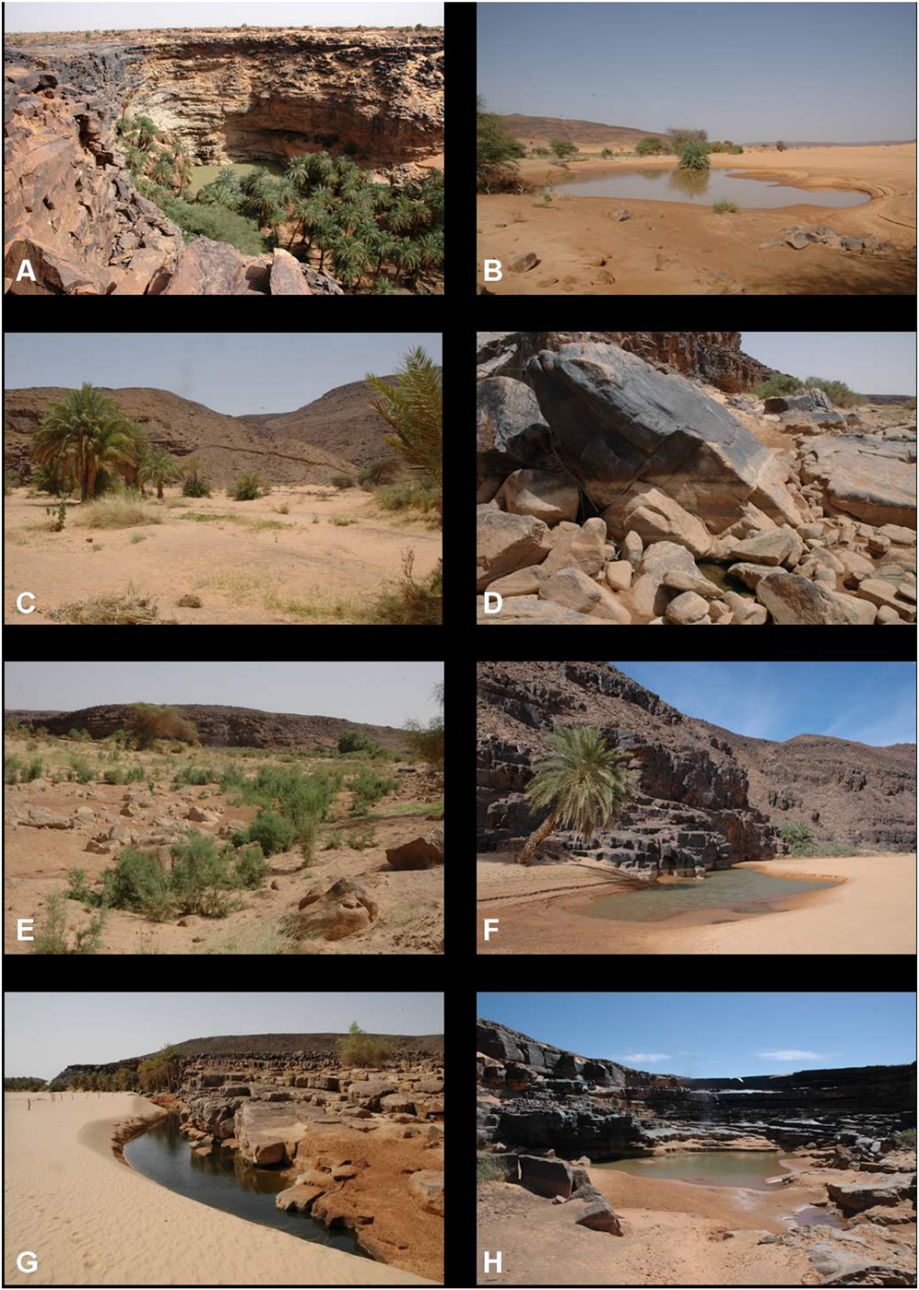
Gueltas and ponds from the Timinit wadi basin. A: El Berbera guelta; B: Ephemeral pond in Timagazine wadi; C: The former perennial pond of Dâyet el Mbârek; D: Agmeïmine guelta; E: The former perennial pond of Dâyet et-Tefla; F: Douâyât Lemgasse pond; G: Dâyet Legleïg pond; H: Nkedeï guelta.

The El Berbera guelta (19°59′181 N, 12°49′374 W) is located in a canyon which opens into the Timinit wadi 4 km further downstream (Figure 3A). Monod observed barbs without being able to capture them and presumed that they were *B. pobeguini* [5].

There were several thousand specimens of *B. pobeguini* in May 2005.

### Timagazine wadi (pool)

In October 1952, Monod observed the presence of *B. pobeguini* in a pool in the Timagazine wadi (upper Timinit wadi) located a little downstream of El Berbera Khenig [6]. The precise location of this pool is uncertain.

No perennial pools were found in the Timagazine wadi downstream of El Berbera Khenig. In September 2007, young *B. pobeguini* were present in a small ephemeral pool 7 km downstream of El Berbera Khenig (19°55′864 N, 12°52′716 W) (Figure 3B). The fish in this pool came undoubtedly from the El Berbera guelta.

### Dâyet el Mbârek (pool)

The Dâyet el Mbârek pool ( = Dhwiyat Lembarrek) is located in the upper Nbéïké wadi at the intersection of two valleys (19°48′720 N, 12°48′459 W). The presence of *B. pobeguini* was reported [5], [6].

This pool, located in a palm grove, was dry in June 2008 (Figure 3C). The residents of the palm grove claimed that it had been perennial until 1990 when it ran dry for the first time, leading to the extinction of the fishes. Since that date, the pool has been largely filled in by sand and only flows for around one month after each flood.

### Agmeïmine (guelta)

The Agmeïmine guelta (19°45′353 N, 12°50′062 W) is located in the bed of the Nbéïké wadi, close to the Doueïr palm grove. The presence of fish in this perennial guelta was not mentioned in the literature.

In December 2007, *B. pobeguini* were present in this guelta and in the temporary Dâyet el Mercrouda pool (19°45′711 N, 12°49′551 W) located 1.3 km upslope in the Doueïr palm grove. In June 2008, the visible surface of the Agmeïmine guelta was only 0.8 m^2^ and most of its volume of water was concealed by large rocks (Figure 3D).

### Dâyet et-Tefla (pool)

The Dâyet et-Tefla pool (19°43′121 N, 12°52′012 W) is located at the foot of a cliff in a meander of the Nbéïké wadi. The presence of *B. pobeguini* was reported [6].

This ancient pool, filled in by alluvial deposits and overgrown with vegetation, was dry in December 2007 and June 2008 (Figure 3E). The L'ehreïjatt guelta (19°43′279 N, 12°51′968 W), located around 300 m upstream, was flowing and home to *B. pobeguini* in December 2007, but was dry in June 2008.

### Douâyât Lemgasse (pool)

The Douâyât Lemgasse pool (19°42′736 N, 12°52′490 W) is located in the bed of the Nbéïké wadi at the foot of a cliff. The presence of fish in this perennial guelta was not mentioned in the literature.

*B. pobeguini* were present in this pool in December 2007 and June 2008, but the water was too cloudy to estimate their abundance. In June 2008 the pool was 1.5 m deep, 60 m in length and 3 m wide (Figure 3F). Around 600 m downstream, the Douâyât Begherbane pool (19°42′445 N, 12°52′534 W) contained *B. pobeguini* in December 2007 but was dry in June 2008.

### Dâyet Legleïb (pool)

The Dâyet Legleïb pool (19°38′654 N, 12°57′873 W) is located in the bed of the Nbéïké wadi, at the intersection of two valleys. The presence of fish in this perennial pool was not mentioned in the literature.

*B. pobeguini* were present in this pool in June 2008 but the water was too cloudy to estimate their abundance. The pool was 1.1 m deep, 62 m in length and 2.5 m wide (Figure 3G).

### Nkedeï (guelta and pool)

**Figure 4:**
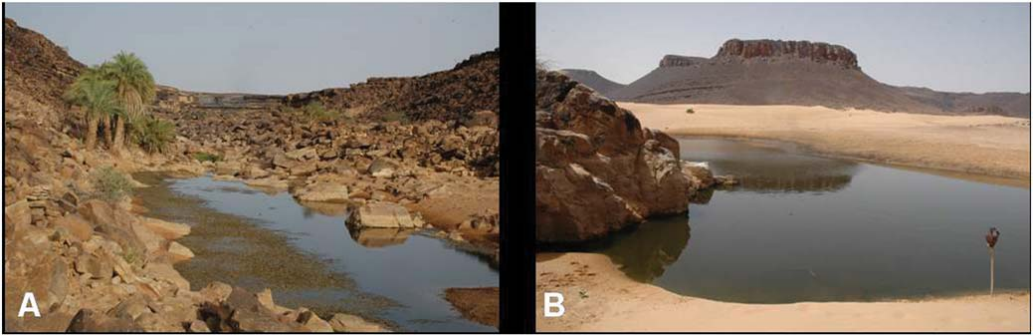
Gueltas and ponds from the Timinit wadi basin (continued). A: Nkedeï pond; B: Glât el Bil pond.

The Nkedeï guelta (19°37′912 N, 12°48′996 W) is at the foot of a cirque cliff (Figure 3H). The Nkedeï pool (19°38′017 N, 12°49′150 W) is located in the bed of the wadi, around 250 m downstream (Figure 4A). The presence of *B. pogeguini* was reported both in the guelta and in the pool [5].

In December 2007, the guelta and the pool provided a habitat for several thousand *B. pobeguini*. In June 2008, the pool was flowing and home to *B. pobeguini* but the guelta was completely dry. Monod [5] claimed that the guelta seemed perennial in the 1950s but it was sometimes reduced to several puddles fed by seepage at the foot of the cliff where the fish congregated.

### Glât el Bil (pool)

Glât el Bil is a large pool located in the bed of the Nkedeï wadi (19°31′498 N, 12°57′636 W), just before it reaches a large sandy plain where in periods of flood the Timinit, Nbéïké and Nkedeï wadis join (Figure 4B). It divides into five parts when the water level falls. The presence of fish in this perennial pool is not mentioned in the literature.

In December 2007 and June 2008 the pool provided a habitat for several thousand *B. pobeguini*. At its largest points in June 2008, it was 110 m in length, 20 m wide and 3.2 m deep.

## Discussion

**Figure 5:**
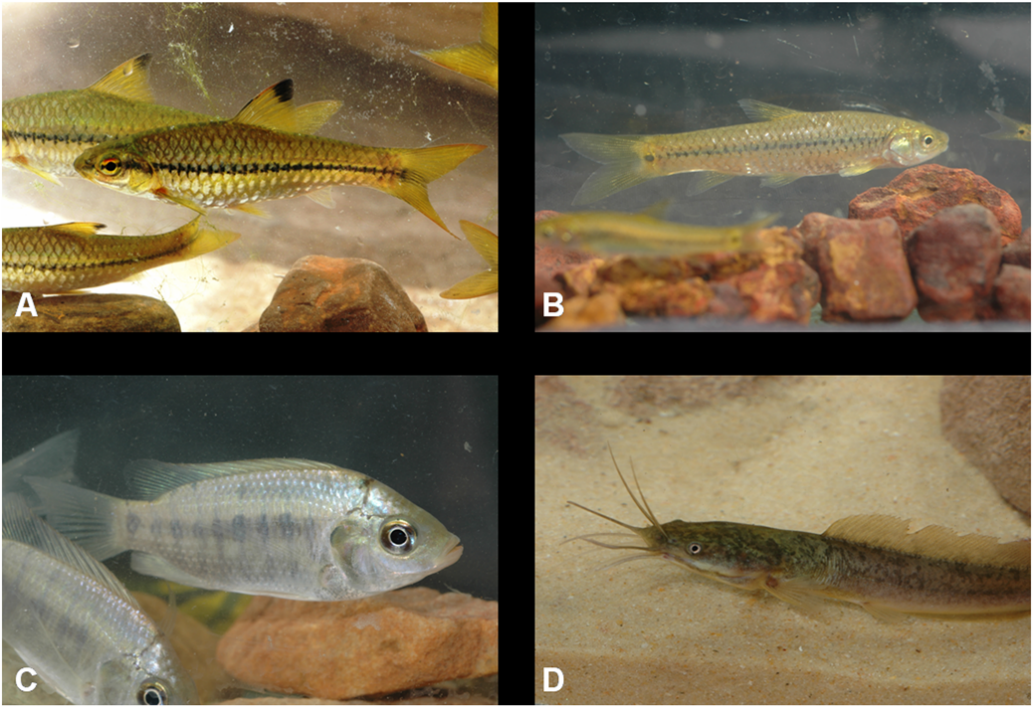
Adrar mountains fishes. A : *Barbus macrops*, Hamdoun guelta ; B : *Barbus pobeguini*, Hamdoun guelta ; C : *Sarotherodon g. galilaeus*, Molomhar guelta ; D : Juvenile *Clarias anguillaris*, Hamdoun guelta.

Four species of fish reported in the Adrar mountains of Mauritania in the 1950s were found in the present study: *B. macrops*, *B. pobeguini*, *S. g. galilaeus* and *C. anguillaris* (Figure 5). However, the population of *B. (Hemigrammocapoeta) mirei* Estève, 1952, which had been described from the Ksar Torchane spring is now extinct. This was the only locality where this species and subgenus were known. Although Monod cast doubt on their validity [6], he nevertheless recognised the specific morphological characteristics (incomplete lateral line) of this population of barbs and recommended further studies in order to understand their origin. With the drying-up of the spring and the extinction of this population, this has now become impossible. The information gathered on the ground clearly indicates that the further deterioration in the rainfall deficit in 1982 and 1983 (19 mm and 7 mm of rainfall in Atar respectively), occurring after twelve years of drought, caused the spring to run dry definitively in early 1984.

Three of the pools and gueltas in the Timinit and Nbéïké wadis where Monod [5], [6] had reported the presence of *B. pobeguini* became ephemeral from 1984 and 1990 onwards, having been perennial before. This was probably due to the reduced underground flow of these wadis which in turn was caused by the ongoing drought. The four pools in the Nbéïké and Nkedeï wadis where the presence of *B. pobeguini* is mentioned for the first time in this study are thought by the populations of the neighbouring oases to have always been perennial. It is likely that the population of *B. pobeguini* for which they provide a habitat is a longstanding one but that it escaped previous investigations.

Of the perennial bodies of water which provide a habitat for fish and which have proved resistant to the ongoing drought, the Hamdoun guelta appears to be the most endangered. In July 2007 its volume was just 1.7 m^3^ with a maximum depth of 35 cm. Two months previously the guelta was still fed by a trickle of water seeping from the length of the cliff wall and providing approximately 0.7 litres of water per minute, but this seep had almost completely dried up in July and was in danger of not compensating for the very high rate of evaporation caused by the heat at this time of the year. With three species of fish present, the Hamdoun guelta presents the second greatest species richness after the Molomhar guelta. The equilibrium maintained between *C. anguillaris* on the one hand, and *B. pobeguini* and *B. macrops* on the other, is noteworthy. The seven adult catfish measuring 22 to 35 cm in length found in the guelta in 2006–07 could easily have killed off the two small populations of barbs - certainly fewer than two hundred individuals - which survived in July 2007. A few pieces of bread thrown into the guelta were immediately fought over by the three species. The rises in water level caused by the rains of August and September 2007, which gave rise to abundant batches of eggs, allowed the barb populations of the guelta to at least double in size within one month (310 specimens counted in September 2007) and to colonise a large number of ephemeral downstream gueltas. The absence of any fish population upstream of the road overlooking the perennial guelta, contrary to observations in the 1950s [5], [12], confirms the increased vulnerability of the Hamdoun guelta. It should also be noted that a single road accident involving one of the many lorries which use this dangerous mountain road could cause a pollution incident which would definitively wipe out the fish population in the guelta.

**Figure 6:**
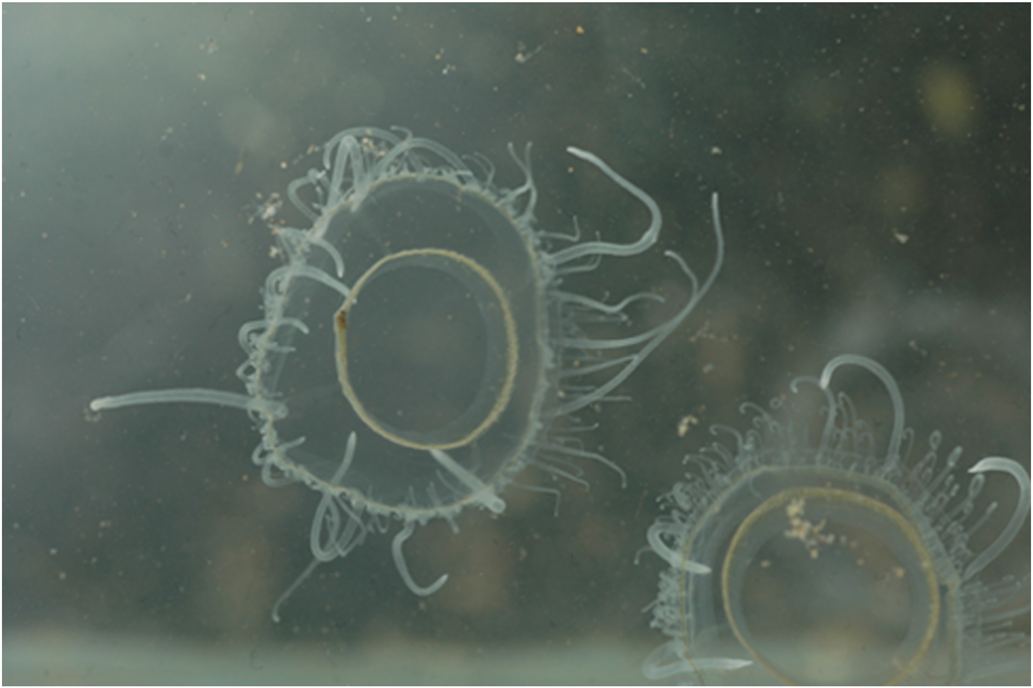
The freshwater jellyfish *Limnocnida tanganyicae*, Molomhar guelta.

The Molomhar gueltas are still the most noteworthy aquatic system in the Adrar region of Mauritania and West Central Sahara. It is the only system which is home to four species of fish, and the only system in the whole central Sahara where *Sarotherodon g. galilaeus* has been able to survive since the Holocene period. Along with the neighbouring Ilîj guelta and several Tibesti gueltas in Chad, it is also one of the few Sahara sites where a freshwater jellyfish, *Limnocnida tanganyicae*, has been observed (Figure 6) [27], [28]. The reduced recorded rainfall does not seem to have affected the dry period water level, given that a similar depth of 6 m in the first guelta had been measured in February 1951 [12]. Two kilometres downstream of the gueltas, at the entrance to the Oumm Lemhar wadi canyon, an underground water harvesting system has been installed in order to provide water for the town of Atar but it does not seem to have had a major impact on the perennial gueltas. Several thousand specimens of *S. g. galilaeus*, *B. pobeguini* and *B. macrops* were present in the gueltas. The size of the population of *C. anguillaris* could not be estimated, but it is certainly much less numerous.

Two large hydrographic networks cover the Adrar region of Mauritania: to the north and west, the Seguellîl wadi network, which occasionally comes into contact with the El Abiod wadi; to the south and east, the Timinit wadi network, into which flow the Nbéïké and Nkedeï wadis. These two large networks were probably separated between 3,500 and 6,000 years before present, and they were also probably in contact with the Senegal River basin in the Holocene and/or Pleistocene periods [14], [29], [30]. During the Holocene, there were large tropical lakes in several regions of Sahara, their whole catchment was probably active, and a diversified fossil fish fauna was collected all over the area [29], [30]. Only *B. pobeguini* have been able to survive in the Timinit wadi basin, perhaps because of high sand-bank and the reduced volume of most of the perennial bodies of water in this basin in dry periods. *B. pobeguini* is a small species (with a maximum total length of 53 mm among 500 adult specimens measured in the Adrar), capable of surviving in a few liters of water.

In 1951, Monod [5] was already underlining the fact that the residual bodies of water in the Sahara constituted an unrivalled natural laboratory, some of them completely isolated representing all that remains of immense but now fossil hydrographical basins. An in-depth genetic comparison of these fish populations with those in the Senegal and Niger basins has yet to be completed. It is currently in progress and should improve our knowledge of when the different basins separated and state precisely whether the levels of divergence reached are compatible with the recognition of distinct specific or subspecific entities.

Despite a drought which has lasted for nearly forty years with an intensity which is without known historical precedent, the relict species of tropical fish in the mountains of the Adrar region of Mauritania continue to survive. However, they appear to be highly endangered. Of the thirteen sites mentioned in the literature, four are no longer home to fish, at least on a permanent basis, and one appears to be highly endangered. In the basin of the Timinit, Nbéïké and Nkedeï wadis, three of the five perennial pools or gueltas previously known to provide a habitat for *B. pobeguini* have become ephemeral. In the Seguellîl wadi basin, the Ksar Torchane spring dried up definitively in 1984, bringing about the extinction of one of the most noteworthy Saharan populations of barb. New fish population extinctions may occur should very low levels of annual recorded rainfall be repeated.
